# Patterns in target-directed breast cancer research

**DOI:** 10.1186/s40064-016-1736-1

**Published:** 2016-02-01

**Authors:** Sofia Torres, Christine Simmons, Jean-François Boileau, Deanna McLeod, Ilidio Martins, Maureen Trudeau

**Affiliations:** Sunnybrook Health Sciences Centre, 2075 Bayview Ave., Room T2 023, Toronto, ON M4N 3M5 Canada; British Columbia Cancer Agency, 600 West 10th Avenue, Vancouver, BC V5Z 4E6 Canada; Jewish General Hospital Segal Cancer Centre, 3755 Côte-Ste-Catherine Road, Montreal, QC Canada; Kaleidoscope Strategic, 146 Marion Street, Toronto, ON M6R 1E7 Canada

**Keywords:** Clinical trials, Target-directed research, Biomarkers, Patient profiling, Randomized trials, Breast cancer

## Abstract

We undertake an analysis of ongoing BC targeted therapy trials registered to CT.gov to describe patterns of ongoing clinical research, highlight gaps in current research programs and identify ways of optimizing ongoing initiatives. A search of clinicaltrials.gov was conducted on September 4, 2013 to identify ongoing randomized phase II and III trials of targeted therapies in BC. A total of 280 trials were analyzed, the majority conducted in either human epidermal growth factor receptor 2 (HER2)-positive (n = 79, 28.2 %) or hormone receptor (HR)-positive (n = 104, 37.1 %) populations. Less than half of all trials were conducted in populations selected to match the agent under investigation (n = 126, 45 %). HER2-directed therapy is the single most investigated class of targeted agents (n = 73, 26.1 %), but trials investigating anti-angiogenic agents are also common (n = 49, 17.5 %). The most common new classes of agents under investigation in HR-positive and triple negative (TN)/BRCA-positive disease, are non-receptor protein kinase-inhibitors (n = 12; 11.5 %) and poly (ADP-ribose) polymerase inhibitors (n = 6; 30 %), respectively. The majority of regimens combine new targeted agents with either chemotherapy (n = 164, 58.6 %) or endocrine therapy (n = 113, 40.4 %); a total of 8 trials (2.8 %) investigated peptide-drug conjugates. The most frequently utilized end-points were pathological complete response in the neo-adjuvant setting (n = 36, 52.9 %) and time-to-event end-points in the adjuvant and advanced settings (77.3 and 72.6 %, respectively). Our findings suggest a need for more target-matched agent development, maintenance of a value-based focus in research and a need for the clinical development of agents to treat TN/BRCA-positive and HR-positive BC.

## Background

Breast cancer (BC) is a significant health concern, with approximately 256,140 new diagnoses of BC in North America annually and 44,720 deaths in 2013 (DeSantis et al. 2013; Canadian Cancer Society’s Steering Committee on Cancer Statistics 2013). Over 600 million dollars are invested in BC research in the United States (US) each year by, the National Cancer Institute alone (National Cancer Institute 2013), and female BC has received the highest allotment of US national expenditure for cancer treatment (National Cancer Institute 2012). For over a decade, a main objective of BC research has been the development of targeted agents designed to improve outcomes while decreasing toxicity (Jain 2014). Efforts to move from a “one size fits all” to a more personalized approach to therapy have resulted in a substantial, multi-faceted body of research. Examples of some of the more significant research gleanings related to trial populations, interventions and trial design are summarized in Table 1. Prominent among these is the discovery of target-matched treatment strategies, the development of targeted treatments in populations enriched for the biological target of interest [e.g., hormone-receptor (HR) or human epidermal growth factor receptor 2 (HER2)]. Recent data show that wide-spread use of target-matched strategies over the last 15 years have resulted in dramatic improvements in the prognosis of patients with estrogen-receptor (ER)-positive (Early Breast Cancer Trialists’ Collaborative Group 2005; Early Breast Cancer Trialists’ Collaborative Group 1998; Davies et al. 2011) and HER2-positive disease (Dawood et al. 2010; Yin et al. 2011; Harris et al. 2011), in both the early and advanced settings. Additionally, the discovery of 6 intrinsic biological BC subtypes (luminal A; luminal B; HER2-enriched; basal-like; normal breast-like; and claudin-low) (Perou et al. 2000; Sorlie et al. 2001; Carey et al. 2006; Prat et al. 2010) has reshaped our understanding of disease biology and shifted our current approach to treatment. Treatment decisions are now guided by prognostic and predictive biomarkers [ER, progesterone receptor (PR) and HER2] which define 3 major therapeutic groups: HER2-positive disease (~20 % of all patients) (Arteaga et al. 2012; Ross et al. 2009), HR-positive disease (~75 %) (Anderson et al. 2011; Lim et al. 2012; Nadji et al. 2005), and triple-negative disease (TN, neither HER2, ER or PR-positive; ~15 %) (Foulkes et al. 2010).

**Table 1:** Lessons learned over the past decade of target-directed research in breast cancer

Lesson	Examples
Trial populations	
Conduct trials in either positively-selected^a^ or target-matched^b^ populations	Identification of 6 intrinsic biological BC subtypes (luminal A; luminal B; HER2-enriched; basal-like; normal breast-like; and claudin-low) (Perou et al. 2000; Sorlie et al. 2001; Carey et al. 2006; Prat et al. 2010) Recurrence scores (e.g. OncotypeDX, PAM50, MammaPrint or IHC4) to help select patients that can forego adjuvant CT (Paik et al. 2006; Albain et al. 2010; Paik et al. 2004; Parker et al. 2009; Chia et al. 2012; Barton et al. 2012; Dowsett et al. 2008; Cuzick et al. 2011; van ‘t Veer et al. 2002; Cardoso et al. 2008; Rutgers et al. 2011; van de Vijver et al. 2002) *Positive trial outcomes* HER2-inhibitors in HER2-positive populations (Slamon et al. 2001; Guan et al. 2013; Goldhirsch et al. 2013; Marty et al. 2005; Perez et al. 2011; Vogel et al. 2002) ET in HR-positive populations (Fisher et al. 1989; Early Breast Cancer Trialists’ Collaborative Group 2005) *Negative trial outcomes* Bevacizumab combinations in HER2-negative populations (Miller et al. 2005, 2007; Miles et al. 2010; Robert et al. 2011; Brufsky et al. 2011) Cetuximab combinations in non-KRAS wild-type (Carey et al. 2012; Baselga et al. 2010; O’Shaughnessy et al. 2007) Inaparib in triple-negative populations (O’Shaughnessy et al. 2011a)
Interventions	
Consider combining T-D with CT	Trastuzumab plus CT (Goldhirsch et al. 2013; Marty et al. 2005; Perez et al. 2011; Slamon et al. 2001; Inoue et al. 2010; Swain et al. 2013) in HER2-positive populations T-DM1 (Verma et al. 2012) in HER2-positive populations
Consider multi-T-D strategies based on a biological rationale	Everolimus plus ET in HR-positive (Baselga et al. 2012b) Dual HER2-inhibition in HER2-positive (Baselga et al. 2012c; Swain et al. 2013; Gianni et al. 2012)
Consider continued T-D therapy	Early setting *Positive trial outcomes* Additional 5 years of tamoxifen (Davies et al. 2013; Gray et al. 2013) or letrozole (Goss et al. 2005) in HR-positive populations *Negative trial outcomes* An additional year of trastuzumab in HER2-positive populations (Goldhirsch et al. 2013) Advanced setting Sequential ET in HR-positive populations (Baselga et al. 2012b) Continued HER2-inhibition in HER2-positive across multiple lines of therapy (Cameron et al. 2008; Verma et al. 2012; von Minckwitz et al. 2009)
Trial design	
Consider the neo-adjuvant setting as a platform for accelerated testing^c^	Pertuzumab (Gianni et al. 2012, 2015), trastuzumab plus FEC and paclitaxel (Buzdar et al. 2013) in HER2-positive NAT populations Trastuzumab plus lapatinib (Baselga et al. 2012a; Robidoux et al. 2012) in HER2-positive patient NAT populations
Utilize phase III trials to arrive at conclusive findings	*Negative trial outcomes* Iniparib in TN populations (O’Shaughnessy et al. 2011a, b) *Positive trial outcomes* The majority of currently established T-D agents (Baselga et al. 2012b, c; Buzdar et al. 1996, 1998; Cameron et al. 2008; Fisher et al. 1989; Slamon et al. 2001; The Nolvadex Adjuvant Trial Organisation 1985; Verma et al. 2012)
Are powered to assess improved survival^d^	*Negative trial outcomes* Bevacizumab combinations in first-line (Miles et al. 2010; Miller et al. 2007; Robert et al. 2011) *Positive trial outcomes* EGF104535 (Guan et al. 2013), CLEOPATRA (Swain et al. 2012; Verma et al. 2012), EMILIA (Baselga et al. 2012c; Swain et al. 2013)

*CT* chemotherapy, *ET* endocrine therapy, *FEC* fluorouracil, epirubicin and cyclophosphamide, *HER2* human epidermal growth factor receptor 2, *HR* hormone receptor, *NAT* neoadjuvant therapy, *OS* overall survival, *pCR* pathological complete response, *T*-*D* target-directed therapy, *T*-*DM1* trastuzumab emtansine, *TN* triple negative

^a^Patient selection is based on over-expression, mutation or other modification of one or more biomarkers or on a multi-biomarker profile/signature with prognostic or predictive value

^b^Biomarker used to positively-select patients is targeted by the investigational T-D agent

^c^Depends on use of pCR as surrogate for survival (pCR translates to disease-free survival and overall survival according to results of the NOAH trial) (Gianni et al. 2013)

^d^Overall survival (or surrogate) as primary end-point

The National Institute of Health’s clinicaltrials.gov (CT.gov) database is the most robust of international trial registries, serving as both a mandatory repository for information on clinical trials conducted under US regulation and a prerequisite for publishing study results in peer-reviewed journals (Hirsch et al. 2013). Although select data are populated by individual investigators and not always consistently reported, the database represents a unique resource through which to evaluate research. The database currently contains detailed information on more than 5000 clinical trials in BC from more than 90 countries (ClinicalTrials.gov 2014b), and ranks BC among the most investigated tumor types per incidence (Hirsch et al. 2013). However, given that clinical research in oncology is both costly and associated with the highest rates of drug attrition and trial failure (Begley and Ellis 2012; Hutchinson and Kirk 2011) we have undertaken an analysis of ongoing BC targeted therapy trials registered to CT.gov to describe patterns of ongoing clinical research, highlight gaps in current research programs and identify ways of optimizing ongoing initiatives.

## Results

### Study selection

**Fig. 1:**
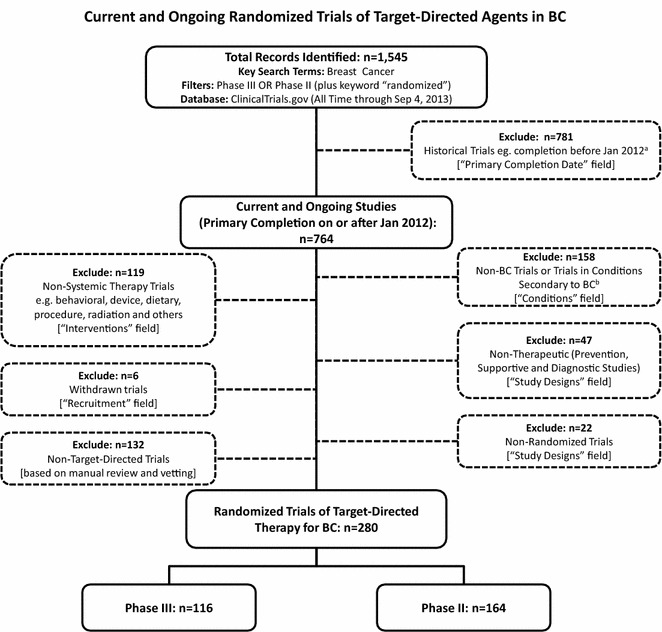
PRISMA diagram representing screening process and final trial eligibility. ^a^Some trial records did not have a primary completion date (n = 246). These trials were deemed not likely to meet the cut-off date and excluded from the database if they met the following criteria: (1) possessed a completion date before January 2012 (n = 112); (2) had completed, terminated or withdrawn status and their records were last verified before January 2012 (n = 37); (3) were not verified by the sponsor in more than 10 years (n = 18); 4) possessed a start date before 1998 (n = 19); ^b^Trials in mixed populations were also excluded. *BC* breast cancer

A total of 1545 matching records were downloaded for analysis, and 1265 studies were excluded (Fig. 1). The remaining data set of 280 trials was locked and parsed to facilitate analysis.

### Populations and classes under development

**Fig. 2:**
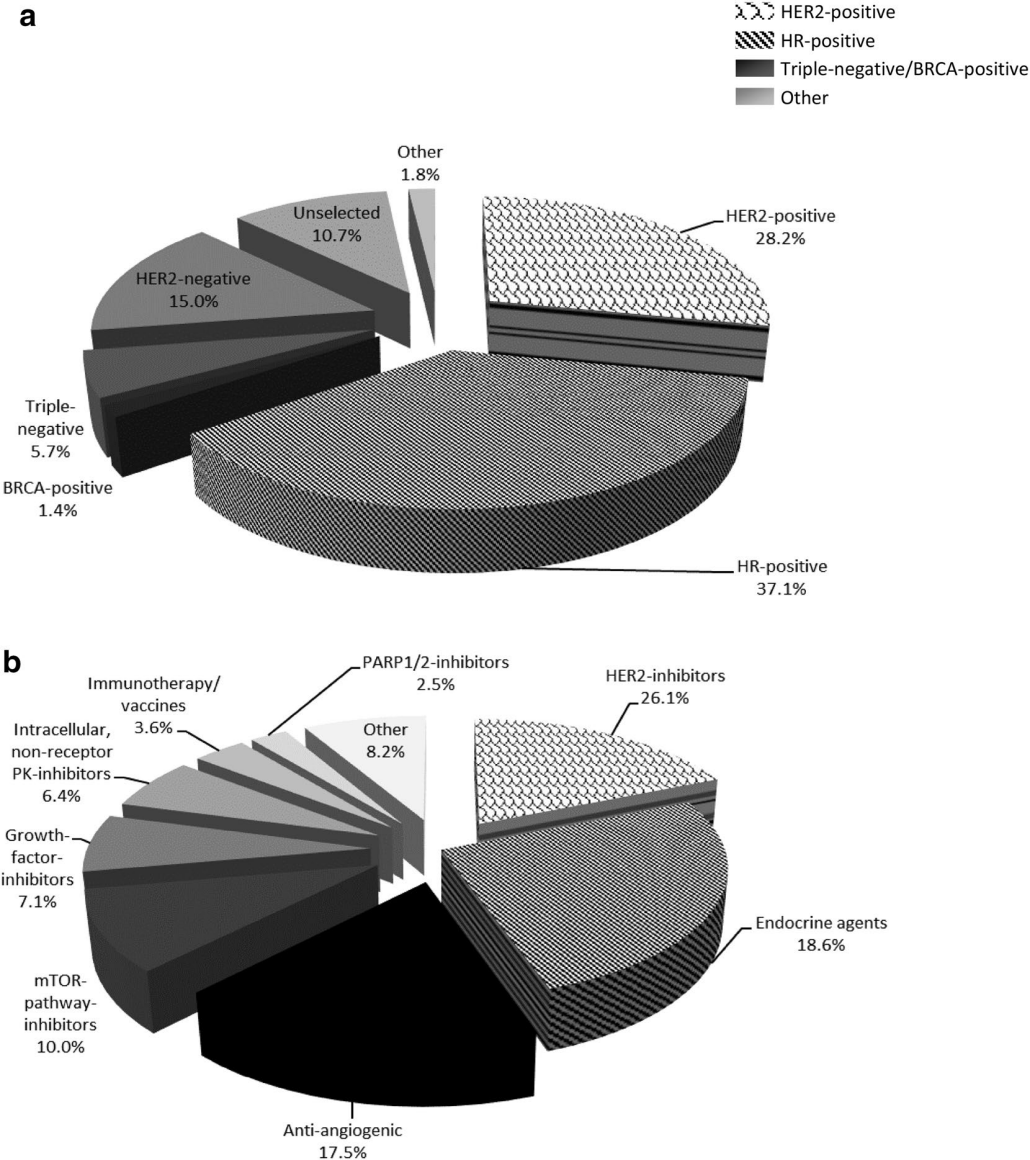
Proportion of different **a** patient subsets, and **b** classes of investigational target-directed agents in current or ongoing trials. *HER2* human epidermal growth factor receptor 2, *HR* hormone receptor, *mTOR* mammalian target of rapamycin, *PARP* poly ADP ribose polymerase, *PK* protein kinase

The majority of trials were conducted in either HER2-positive (n = 79, 28.2 %) or HR-positive (n = 104, 37.1 %) populations (Fig. 2a). Trials conducted in TN/BRCA-positive disease accounted for 7.1 % (n = 20) of all research. Trials in all other populations accounted for 27.5 % (n = 77) of research. Less than half of all ongoing trials were conducted in target-matched (i.e., enriched for the biological target of the therapy under investigation) populations (n = 126, 45.0 %; Table 2). The most investigated classes of agents were HER2-inhibitors (n = 73, 26.1 %), endocrine agents (n = 52, 18.6 %) and anti-angiogenic agents (n = 49, 17.5 %; Fig. 2b). The proportion of research dedicated to the development of emergent agents was only slightly greater than the proportion addressing established agents (52.5 and 47.5 %, respectively), and consisted mostly of phase II studies (74.8 %).

**Table 2:** Randomized trial characteristics by biological subtype of trial population and treatment setting

Categories	HER2-positive [n (%)]	HR-positive [n (%)]	Triple-negative or BRCA-positive [n (%)]	Other or unselected [n (%)]	Total [n (%)]
Total, n (proportion by subtype, %)	79 (28.2)	104 (37.1)	20 (7.1)	77 (27.5)	280 (100.0)
Populations					
Target-matched	68 (86.1)	50 (48.1)	1 (5.0)	7 (9.1)	126 (45.0)
Non target-matched	11 (13.9)	54 (51.9)	19 (95.0)	70 (90.9)	154 (55.0)
Investigational target-directed classes					
HER2-inhibitors	64 (81.0)	4 (3.8)	0 (0)	5 (6.5)	73 (26.1)
Endocrine agents	0 (0)	47 (45.2)	1 (5.0)	4 (5.2)	52 (18.6)
Anti-angiogenics	5 (6.3)	7 (6.7)	4 (20.0)	33 (42.8)	49 (17.5)
mTOR/PI3K/Akt pathway-inhibitors	4 (5.1)	14 (13.5)	3 (15.0)	7 (9.1)	28 (10.0)
Growth factor-inhibitors	2 (2.5)	12 (11.5)	2 (10.0)	4 (5.2)	20 (7.1)
Intracellular, non-receptor PK-inhibitors	0 (0)	12 (11.5)	1 (5.0)	5 (6.5)	18 (6.4)
Immunotherapy/vaccines	4 (5.1)	0 (0)	0 (0)	6 (7.8)	10 (3.6)
PARP1/2-inhibitors	0 (0)	1 (1.0)	6 (30.0)	0 (0)	7 (2.5)
Other	0 (0)	7 (6.7)	3 (15.0)	13 (16.9)	23 (8.2)
Types of target-directed therapy					
Established	59 (74.7)	56 (53.8)	2 (10.0)	16 (20.8)	133 (47.5)
Emergent	20 (25.3)	48 (46.2)	18 (90.0)	61 (79.2)	147 (52.5)
Therapeutic strategies					
Chemotherapy-based regimens	64 (81.0)	13 (12.5)	18 (90.0)	69 (89.6)	164 (58.6)
Non chemotherapy-based regimens	15 (19.0)	91 (87.5)	2 (10.0)	8 (10.4)	116 (41.4)
ET-based regimens	6 (7.6)	95 (91.3)	1 (5.0)	11 (14.3)	113 (40.4)
Non ET-based regimens	73 (92.4)	9 (8.6)	19 (95.0)	66 (85.7)	167 (59.6)
Peptide-drug conjugates	6 (7.6)	0 (0)	1 (5.0)	1 (1.3)	8 (2.8)
Mono-class regimens	60 (75.9)	51 (49.0)	19 (95.0)	65 (84.4)	195 (69.6)
Multi-class regimens	19 (24.0)	53 (51.0)	1 (5.0)	12 (15.6)	85 (30.4)

Categories	Neoadjuvant [n (%)]	Adjuvant [n (%)]	Advanced [n (%)]	Total [n (%)]
Total [n (proportion by setting, %)]	68 (24.3)	66 (23.6)	146 (52.1)	280 (100.0)
Subtype				
HER2-positive	25 (36.8)	19 (28.8)	35 (23.9)	79 (28.2)
HR-positive	17 (25.0)	29 (43.9)	58 (39.7)	104 (37.1)
Triple-negative or BRCA-positive	7 (10.3)	3 (4.5)	10 (6.8)	20 (7.1)
Other or unselected	19 (27.9)	15 (22.7)	43 (29.4)	77 (27.5)
Primary endpoint				
Overall survival	0 (0)	1 (1.5)	6 (4.1)	7 (2.5)
Quality of life	0 (0)	2 (3.0)	1 (0.7)	3 (1.1)
Pathological complete response	36 (52.9)	0 (0)	0 (0)	36 (12.8)
DFS/RFS/PFS/EFS	3 (4.4)	51 (77.3)	106 (72.6)	160 (57.1)
Clinical response	15 (22.0)	1 (1.5)	19 (13.0)	35 (12.5)
Biomarker	10 (14.7)	6 (9.1)	2 (1.4)	18 (6.4)
Safety and tolerability	4 (5.9)	3 (4.5)	8 (5.5)	15 (5.4)
Other	0 (0)	2 (3.0)	4 (2.7)	6 (2.1)
Study Phase				
Phase II (%)	53 (77.9)	15 (22.7)	96 (65.8)	164 (58.6)
Phase III (%)	15 (22.0)	51 (77.3)	50 (34.2)	116 (41.4)

*DFS* disease-free survival, *EFS* event-free survival, *ET* endocrine therapy, *HER2* human epidermal growth factor receptor 2, *HR* hormone receptor, *mTOR* mammalian target of rapamycin, *PARP* poly(ADP-ribose) polymerase, *PI3K* phosphoinositide 3-kinase, *PFS* progression-free survival, *RFS* relapse-free survival

### Therapeutic strategies

The majority of regimens under investigation combined new targeted agents with either chemotherapy (n = 164, 58.6 %) or endocrine therapy (ET, n = 113, 40.4 %; Table 2). A total of 8 trials (2.8 %) investigated peptide-drug conjugates, six trials assessed the HER2 antibody–drug conjugate ado-trastuzumab-emtansine (T-DM1) in HER2-positive disease, one trial tested a luteinizing-hormone-releasing hormone receptor (LHRH-R)-antibody conjugate in TN disease (LHRH-R-positive) and one investigated a glycoprotein NMB (GpNMB)-directed conjugate in a population selected for GpNMB expression.

**Fig. 3:**
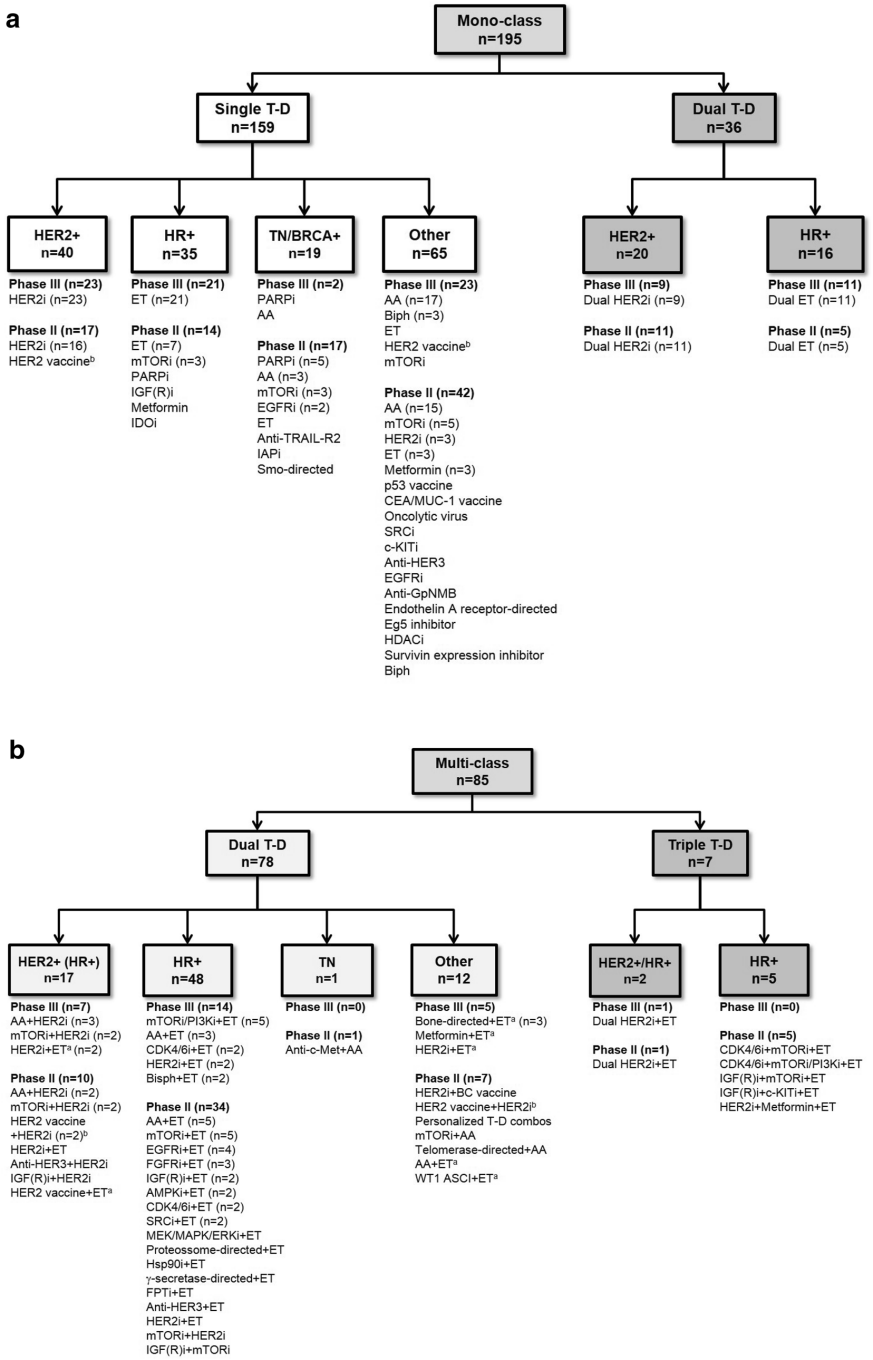
Flowchart of trials with **a** one or **b** multiple classes of target-directed agents in the investigational arm. ^a^One of the standard therapy options possible. In early (adjuvant and/or neoadjuvant) trials, standard therapy (including ET) may have been required before, during or after the investigational treatment and either before or after surgery. ^b^Trials of HER2 vaccines that included patients with high levels (overexpression) of HER2 (in addition to patients with low and intermediate HER2 levels) were categorized under “HER2-positive” while those that did not include patients overexpressing HER2 were categorized under “Other”. *Note*: When present, non-T-D (including chemotherapy) agents were omitted from the investigational regimen short description. *AA* anti-angiogenic, *AMPKi* AMPK inhibitor, *ASCI* Antigen-Specific Cancer Immunotherapeutic, *Bisph* bisphosphonates, *BRCA* + BRCA-positive, *CDK4/6i* cyclin-dependent kinase 4/6 inhibitor, *c*-*KITi* c-KIT (and BCR/Abl and Src or PDGFR) inhibitor, *dual T*-*D* experimental regimens containing two target-directed agents, *EGFRi* epidermal growth factor receptor inhibitor, *ET* endocrine therapy, *FGFRi* fibroblast growth factor receptor inhibitor, *FPTi* farnesyl protein transferase inhibitor, *GpNMB* glycoprotein NMB, *HDACi* histone deacetylase inhibitor, *HER2* + human epidermal growth factor receptor-positive, *HER2i* human epidermal growth factor receptor inhibitor, *HR* + hormone receptor-positive, *Hsp90i*, heat shock protein 90 inhibitor, *IAPi* inhibitor of apoptosis inhibitor, *IDOi* IDO pathway inhibitor, *IGF(R)i* insulin growth factor (receptor) inhibitor, *MEK/MAPK/ERKi* MEK or MAPK/ERK inhibitor, *mono*-*class* experimental regimens containing only one class of target-directed agents, *mTORi* mammalian target of rapamycin pathway inhibitor, *multi*-*class* experimental regimens containing more than one class of target-directed agents, *PARPi* poly(ADP-ribose) polymerase inhibitor, *PI3Ki* phosphoinositide 3-kinase inhibitor, *Smo* smoothened, *SRCi* SRC kinase family inhibitor, *T*-*D* target-directed therapy, *TN* triple-negative

A broad range of therapeutic strategies were tested, with most trials investigating a single class of agents (mono-class, n = 195, 69.6 %; Table 2), either used alone (single-targeted, n = 159, 81.5 %, with or without non-target-directed therapy; Fig. 3a) or in combination with agents from the same class (dual-targeted, n = 36, 18.5 %). Of the trials investigating targeted combinations from different classes (multi-class, n = 85, 30.4 %; Table 2), most combined two agents (dual-targeted, n = 78, 91.8 %; Fig. 3b) and others combined three agents (triple-targeted, n = 7, 8.2 %).

### HER2-positive

In HER2-positive disease, HER2-inhibitor trials made up 81.0 % (n = 64) of ongoing research, while other research was directed toward anti-angiogenics (n = 5, 6.3 %), mammalian target of rapamycin (mTOR)/phosphoinositide 3-kinase (PI3K)/protein kinase B (Akt)-inhibitors (n = 4, 5.1 %) and immunotherapy/vaccines (n = 4, 5.1 %; Table 2). Mono-class trials (n = 60, 75.9 %; Fig. 3a) employed either a single HER2-inhibitor approach (n = 39, 65.0 %) or a dual-HER2-inhibitor approach (n = 20, 33.3 %), with the exception of a single HER2 vaccine trial (n = 1, 1.6 %). Multi-class trials (n = 19, 24.1 %; Fig. 3b) were generally characterized by HER2-directed therapy combined with either anti-angiogenics (n = 5, 26.3 %), ET (n = 5, 26.3 %) or mTOR-inhibitors (n = 4, 21.0 %). These trials included two conducted in HER2/HR co-positive populations: one combining ET with a dual HER2-blockade and another combining ET with a HER2-inhibitor and a HER2 vaccine.

### HR-positive

In HR-positive disease, ET made up 45.2 % (n = 47; Table 2) of ongoing research, and 13.5 % was focused on mTOR/PI3K/Akt-inhibitors (n = 14). Other classes under investigation in this area were intracellular, non-receptor protein kinase (PK)-inhibitors (n = 12, 11.5 %) and growth factor-inhibitors (n = 12, 11.5 %). Mono-class trials (n = 51, 49.0 %; Fig. 3a) investigating ET therapy (n = 44, 86.3 %) or mTOR pathway-inhibitor therapy (n = 3, 5.9 %) were common. Dual-targeted approaches combined traditional ET, such as tamoxifen- or aromatase-inhibitors with LHRH-R-agonists (n = 10) or androgen receptor (AR)-targeted agents (n = 1). Multi-class trials (n = 53, 51.0 %; Fig. 3b) commonly comprised ET in combination with either mTOR/PI3K-inhibitors (n = 10, 18.9 %), anti-angiogenics (n = 8, 15.1 %) or cyclin-dependent kinase 4 and 6 (CDK4/6)-inhibitors (n = 4, 7.5 %). A small number of trials also explored a triple-targeted approach (n = 5, 9.4 %), combining CDK4/6-inhibitors plus mTOR/PI3K-inhibitors and ET (n = 2), IGF(R)-inhibitors plus either a c-KIT- or mTOR-inhibitor and ET (n = 2), or a HER2-inhibitor plus metformin and ET (n = 1).

### Triple-negative/BRCA-positive

In TN/BRCA-positive disease, poly(ADP-ribose) polymerase (PARP) 1/2-inhibitors were the most studied class of drugs (n = 6, 30.0 %; Table 2) followed by anti-angiogenics (n = 4, 20.0 %) and mTOR/PI3K/Akt-inhibitors (n = 3, 15.0 %). Mono-class trials (n = 19, 95.0 %; Fig. 3a) focused on PARP-inhibitors (n = 6, 31.6 %), mTOR-inhibitors (n = 3, 15.8 %) and anti-angiogenics (n = 3, 15.8 %). Trials combining multiple classes of agents (n = 1, 5.0 %; Fig. 3b) were less prevalent, with only one combining a c-met-inhibitor and an anti-angiogenic agent.

### Other

In other populations, anti-angiogenics remained a key area of research (n = 33, 42.8 %; Table 2). Mono-class trials (n = 65, 84.4 %; Fig. 3a) focused predominantly on anti-angiogenic agents (n = 32, 49.2 %), while some research explored mTOR-inhibitors (n = 6, 9.2 %), HER2-inhibitors (n = 3, 4.6 %), ET (n = 3, 4.6 %), bisphosphonates (n = 3, 4.6 %), and metformin (n = 3, 4.6 %). Some multi-class research was ongoing (n = 12, 15.6 %; Fig. 3b), specifically combining bone-directed therapy and ET (n = 3, 25 %). Trials of HER2-inhibitors and/or vaccines were also conducted in HER2-negative patients (n = 6), including those with low or intermediate levels of HER2 and/or with HER2-expressing disseminated tumor cells.

### Setting, primary end-points and trial design

**Fig. 4:**
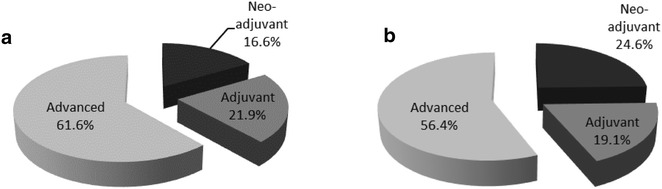
Frequency of neoadjuvant, adjuvant and advanced studies in trials with primary completion date of **a** 2007–2011 or **b** 2012–2016

The majority of ongoing target-directed research was conducted in the advanced setting (n = 146, 52.1 %), with fewer studies in the neo-adjuvant (n = 68, 24.3 %) and adjuvant (n = 66, 23.6 %) settings (Table 2). In the neo-adjuvant setting, most research was conducted in HER2-positive disease (n = 25, 36.8 %), while only 10.3 % (n = 7) was conducted in TN/BRCA-positive populations. Both the total number and proportion of trials conducted in the neo-adjuvant setting in the 5-year period beginning January 2012 increased compared with those of the preceding 5-year period (2007–2011, n = 25, 16.6 % vs 2012–2016, n = 58, 24.6 %; Fig. 4). The majority of trials in the adjuvant and advanced settings involved HR-positive (n = 29, 43.9 % and n = 58, 39.7 %, respectively; Table 2) and HER2-positive populations (n = 19, 28.8 % and n = 35, 23.9 %, respectively).

The primary end-points used in targeted trials varied by setting (Table 2). In the neo-adjuvant setting, the most common end-points were pathological complete response (pCR; n = 36, 52.9 %), clinical response (n = 15, 22.0 %) and biomarker measurement (n = 10, 14.7 %). In the adjuvant and advanced settings, time-to-event end-points were common (77.3 and 72.6 %, respectively) while the use of overall survival as a primary end-point in any setting was rare (1.5 and 4.1 %, respectively).

There was a slightly greater proportion of phase II trials compared with phase III trials overall (n = 164, 58.6 %; Table 2). Phase II trials were most common in the neo-adjuvant (n = 53, 77.9 %) and advanced (n = 96, 65.8 %) settings, while phase III trials were more common in the adjuvant setting (n = 51, 77.3 %).

## Discussion

### Populations

Given the prevalence of expression and demonstrated ability to target HER2 and HRs, the proportion of research dedicated to populations defined by these biomarkers is appropriate. However, relative to the overall incidence of HR-positive and TN disease (~75 % (Lim et al. 2012; Anderson et al. 2011; Nadji et al. 2005) and ~15 % (Foulkes et al. 2010)), the amount clinical development in these settings is low and highlights a need for further research in these settings.

### Elimination of chemotherapy

One of the great promises of targeted therapy was the potential to reduce or eliminate the need for chemotherapy and its indiscriminate effect on normal tissue. However, after more than a decade of research, the majority of trials conducted in non-HR-positive populations (n = 151, 85.8 %) combine targeted agents with chemotherapy. It is only recently that trials have begun to explore the removal of chemotherapy from targeted regimens for select populations; e.g., eliminating chemotherapy from HER2-directed regimens in elderly adjuvant patients (n = 1) or from dual HER2-targeted combinations in the advanced and neo-adjuvant settings (n = 2).

Recurrence scores based on clinicopathological features [e.g., Nottingham Prognostic Index (Blamey et al. 2007), Adjuvant! (Ravdin et al. 2001), and PREDICT (Wishart et al. 2011)] have also been useful in identifying patients who may forego adjuvant chemotherapy. Gene signature-based scores are now being validated in randomized phase III trials in intermediate-risk, HR-positive patients (TAILORx (ClinicalTrials.gov 2014a), RxPONDER (ClinicalTrials.gov 2014d), and MINDACT (ClinicalTrials.gov 2013)), and are expected to further define subsets of patients who may be spared the toxicity of chemotherapy (Viale et al. 2014; Bogaerts et al. 2006). Antibody-cytotoxic conjugates are yet another important means by which chemotherapy-associated adverse effects can be reduced. In HER2-positive disease, a HER2-directed cytotoxic is replacing existing single-agent targeted therapy or cytotoxic-targeted combinations, and a GpNMB-directed cytotoxic is being developed for GpNMB-expressing TN disease (METRIC trial, NCT01997333) (Celldex Therapeutics 2012; ClinicalTrials.gov 2012, 2014c).

### Combinatorial strategies

Effectively targeting oncogenic mutations or copy number alterations has proven challenging, with no new agents identified in the last 15 years. In this context, combinatorial approaches have become one of the most commonly explored strategies. In HER2-positive disease, mono-class regimens combining multiple targeted agents to more effectively block a given receptor have become the focus of ongoing combinatorial research. Dual-HER2-inhibition has held much promise in both the advanced (Baselga et al. 2012c; Verma et al. 2012) and neo-adjuvant (Gianni et al. 2012) settings, yet findings from the ALTTO trial, showing a lack of improvement with the addition of lapatinib to standard adjuvant targeted therapy, calls into question the benefits of this approach in earlier settings (Piccart-Gebhart et al. 2014). Results from the APHINITY trial (NCT01358877), assessing the addition of pertuzumab (rather than lapatinib) to adjuvant targeted therapy, will help clarify the role for combinatorial strategies in early disease.

In HR-positive disease, a main direction of research has been the development of multi-class regimens to inhibit secondary processes, such as treatment resistance (mTOR/PI3K-inhibitors; insulin growth factor receptor [IGF(R)]-inhibitors; epidermal growth factor receptor [EGFR]-inhibitors; fibroblast growth factor receptor [FGFR]-inhibitors), cell cycle regulation (CDK4/6-inhibitors) or effects of the tumor micro-environment (bisphosphonates). Breakthroughs such as the addition of the mTOR-inhibitor everolimus to exemestane in advanced BC resistant to prior non-steroidal aromatase-inhibitor therapy (Baselga et al. 2012b) and the addition of palbociclib to fulvestrant in patients with advanced BC progressing on prior ET therapy (Turner et al. 2015) illustrate the promise of combinatorial approaches in enhancing established targeted strategies. However, questions of tolerability and cost remain as combinatorial strategies are undertaken to more completely inhibit pro-oncogenic pathways.

### Neo-adjuvant setting: platform for accelerated drug development

The neo-adjuvant setting provides a unique platform for targeted agent research, with opportunities for correlative studies and the potential for translating discovery into benefit in the adjuvant setting. Relative to drug development, improvements in pCR have been correlated with survival outcomes in HER2-positive and TN subtypes (Cortazar et al. 2014) and can be used as the basis for accelerated FDA approval (Prowell and Pazdur 2012). Although the FDA approved pertuzumab in the neoadjuvant setting, the results of the Neosphere trial did not show a statistically significant association between pCR and 3-year disease-free survival and progression-free survival (PFS) (Gianni et al. 2015). The increase in both number and proportion of clinical trials conducted in this setting over the last several years suggests an increased commitment to neo-adjuvant research, although data also suggests that it remains an underutilized strategy.

The negative results of the ALTTO study (Piccart-Gebhart et al. 2014), evaluating an adjuvant dual-HER2-blockade, bring into question the assumption that benefits in the neo-adjuvant setting (Piccart-Gebhart et al. 2013) automatically translate into adjuvant benefits. These findings underscore the complexity and challenges of accelerated drug development. Innovative approaches to neo-adjuvant research, using adaptive Bayesian designs and pCR as the primary end-point, to rapidly select active novel agents (e.g., ISPY2 trial (ClinicalTrials.gov 2015)), may lead to more efficient use of research resources by requiring fewer patients, although absolute magnitudes of benefit are difficult to assess using this type of approach and results require phase III confirmation.

### Optimization of research resources

Although BC is the most investigated disease site (Hirsch et al. 2013), it is also an area of research associated with one of the highest rates of drug attrition and trial failure (Begley and Ellis 2012; Hutchinson and Kirk 2011). Presently, anti-angiogenic agent trials (n = 49, 17.5 %) comprise almost a fifth of all ongoing research, and the total number of these trials is comparable the sum of all ET directed research (n = 52, 18.6 %). Clinical testing of anti-angiogenics in BC has been marked by failure to demonstrate clinically significant PFS and survival benefits and an increased risk of serious side effects (Miles et al. 2010; Robert et al. 2011; Hamburg 2011; Barrios et al. 2010; Baselga et al. 2012d; Mackey et al. 2013). Despite this, as of September 2013, a total of 25,784 patients were accrued to current anti-angiogenic trials, with planned accrual of an additional 3833 patients across 12 trials. A 2006 survey of leading developers estimates that the cost of enrolling a patient into a phase III trial is $26,000 (lifesciences world 2006; Stewart et al. 2010). Given these figures, the investment directed toward anti-angiogenic research has amounted to a staggering $770,042,000. As the hope of success continues to entice patients and clinicians alike to fully explore the benefits of a given class of therapy, prudence would call for a redirection of resources towards classes of agents that have demonstrated therapeutic benefit or for which a biomarker is available to guide therapy. This is best exemplified in the recent discovery of the 14-gene signature to identify immune-enriched patients who preferentially respond to trastuzumab therapy (Perez et al. 2014).

## Conclusions

Target-directed research is essential to ongoing research efforts in BC and our understanding of how to optimize these strategies continues to evolve. Our findings suggest that there is a continued need for target-matched agent development, maintenance of a value-based focus in research and a need for the clinical development of agents to treat TN/BRCA-positive and HR-positive BC.

## Methods

### Target-directed trial dataset

A search of the CT.gov website was conducted on September 4, 2013 to identify randomized phase II and III trials of targeted therapies in BC. We considered targeted therapies to be anti-cancer drugs with a clear cellular or molecularly-directed mechanisms of action that interfere with cell growth signaling or tumor blood vessel development, promote death of specific cell types, or stimulate the immune system to destroy specific cell types and/or deliver toxic drugs to cancer cells (National Cancer Institute 2014). All non-randomized, non-systemic, non-therapeutic, or withdrawn trials, as well as those conducted in a non-invasive setting, without a target-directed agent in the experimental arm, or with a primary completion date (date of primary outcome data collection, or date expected) before January 2012, were excluded.

### Trial review and classification

Each trial was classified and analyzed based on the following 9 criteria, which were established based on the record title: (1) degree to which the investigational targeted agent is established (defined below), (2) number and (3) class of targeted agents in the investigational arm, (4) use of continued targeted therapy, (5) setting, (6) biological subtype of population, (7) status of trial, (8) study type and (9) end-points used. If the category was unclear, conditions and key words were assessed or the full CT.gov record was reviewed.

Established targeted-drugs, defined as those with at least one US Food and Drug Administration (FDA)-approved BC indication as of September 4, 2013, are summarized in Table 3. Bone-modifying/remodeling agents and progesterone were considered established due to their historical and widespread use in BC treatment; all other agents were defined as emergent. Trials were categorized into 4 mutually exclusive groups based on the biomarker status of the trial population, in order of therapeutic relevance, as follows: HER2-positive; HR-positive; TN or BRCA-mutated; (TN/BRCA-positive); and other populations (HER2-negative trials with HR status unspecified; other subtypes and unselected; not defined by biomarker status).

**Table 3:** Target-directed agents classified by class of agent and whether established or emergent

Drug class and description	Established	Emergent
Anti-angiogenic therapy: Drugs that interfere with angiogenesis and block tumor growth (target VEGF, VEGF receptor or block kinases involved in VEGF signaling)		Bevacizumab, Cabozantinib, Endostar, Icrucumab, Nintedanib (BIBF 1120), Pazopanib, Ramucirumab, Sorafenib, Sunitinib, Tivozanib, Trebananib (AMG 386), Vandetanib
HER2 targeted therapy: Drugs that bind to Her2 or inhibit its tyrosine-kinase activity	Lapatinib, Pertuzumab, T-DM1, Trastuzumab	Afatinib, AZD8931, Neratinib, Trastuzumab biosimilars (ABP 980 and BCD-022)
Growth factor-inhibitors: Drugs that bind to EGFR, HER3, HER4, IGFR, FGFR, PDGFR and RANKL or inhibit the tyrosine-kinase activity of these receptors	Denosumab	AZD4547, BMS-754807, Cetuximab, Cixutumumab, Dalotuzumab, Dovitinib, Erlotinib, Ganitumab, Gefitinib, Imatinib, MEDI-573, MM-121, U3-1287
mTOR/PI3K/Akt-pathway-inhibitors: Drugs that inhibit signaling of the pathway	Everolimus	AZD5363, BEZ235, BKM120, BYL719, DLBS1425, GDC-0941, GDC-0980, MK-2206, PF-4691502, Ridaforolimus
Therapies that target ER or hormonal production: Drugs that interfere with estrogen/androgen ability to promote tumor growth and proliferation	Anastrozole, Exemestane, Fulvestrant, Goserelin (ZD9393), Letrozole, Leuprorelin, Progesterone, Tamoxifen, Toremifene, Triptorelin	Abiraterone, AEZS-108, CDB-4124, Irosustat
PARP1/2-Inhibitors: Drugs that inhibit of the activity of PARPs.For historical reasons, iniparib was included in this class		Iniparib, Niraparib, Rucaparib, Veliparib (ABT-888)
Intracellular, non-receptor PK-inhibitors: Includes inhibitors of Aurora A kinase, CDK4-6, c-Met, Src-family, MEK/MAPK/ERK and AMPK		Alisertib (MLN8237), AZDO530, Dasatinib, LEE011, Metformin, Onartuzumab, Palbociclib (PD-0332991), Selumetinib
Immunotherapy/cancer vaccines: Drugs that target the immune system to destroy cancer cells or interfere with growth of specific cancer cells		Allogeneic GM-CSF-secreting breast cancer vaccine, autologous dendritic cell-adenovirus p53 vaccine, GSK2302024A, HER-2/neu peptide vaccines, Ipilimumab, PANVAC-V/F, Reolysin
Antibody–drug conjugates: Drugs composed of an targeted drug (antibody) and a cytotoxic drug, delivered only to the targeted cancer cell	T-DM1	AEZS-108, Glembatumumab vedotin
Other targeted therapies: Drugs with other targets not included previously or trials that include multiple targeted therapies	Biphosphonates (Ibandronate, Zoledronate)	Bortezomib, Erismodegib (LDE225), Ganetespib, Imetelstat, Indoximod, LCL161, Litronesib, RO4929097, Tigatuzumab, Tipifarnib, YM155, Zibotentan (ZD4054)

*CDK* cyclin-dependent kinase, *EGFR* epidermal growth factor receptor, *ER* estrogen receptor, *HER2* human epidermal growth factor receptor 2, *HER3* human epidermal growth factor receptor 3, *HER4* human epidermal growth factor receptor, *IGFR* insulin-like growth factor receptor, *FGFR* dibroblast growth factor receptor, *mTOR* mammalian target of rapmycin, *PARP* poly(ADP-ribose) polymerase, *PI3K* phosphoinositide 3-kinase, *PDGFR* platelet-derived growth factor receptor, *RANKL* receptor activator of nuclear factor kappa-B ligand, *T*-*DM1* trastuzumab emtansine, *VEGF* vascular endothelial growth factor

To assess the degree to which neo-adjuvant trials have changed over the last 5 years, trials with primary completion dates between 2012 and 2016 were compared to those with primary completion dates between 2007 and 2011.

## Abbreviations

Akt: protein kinase B
AR: androgen receptor
BC: breast cancer
CT.gov: clinicaltrials.gov
CDK: cyclin-dependent kinase
EGFR: epidermal growth factor receptor
ER: estrogen receptor
ET: endocrine therapy
FDA: US Food and Drug Administration
FGFR: fibroblast growth factor receptor
GpNMB: glycoprotein NMB
HER2: human epidermal growth factor receptor 2
HR: hormone-receptor
IGFR: insulin growth factor receptor
LHRH-R: luteinizing-hormone-releasing hormone receptor
mTOR: mammalian target of rapamycin
PARP: poly(ADP-ribose) polymerase
pCR: pathological complete response
PI3K: phosphoinositide 3-kinase
PR: progesterone receptor
PK: protein kinase
T-DM1: ado-trastuzumab-emtansine
TN: triple-negative
US: United States
